# The Autophagy-Lysosomal Pathways and Their Emerging Roles in Modulating Proteostasis in Tumors

**DOI:** 10.3390/cells8010004

**Published:** 2018-12-20

**Authors:** Zhen Dong, Hongjuan Cui

**Affiliations:** 1 State Key Laboratory of Silkworm Genome Biology, Southwest University, Beibei, Chongqing 400716, China; zdong007@swu.edu.cn; 2 Engineering Research Center for Cancer Biomedical and Translational Medicine, Southwest University, Beibei, Chongqing 400716, China; 3 Chongqing Engineering and Technology Research Center for Silk Biomaterials and Regenerative Medicine, Southwest University, Beibei, Chongqing 400716, China; 4 Institute of Sericulture and Systems Biology, Southwest University, Beibei, Chongqing 400716, China

**Keywords:** autophagy-lysosomal pathways, proteostasis, protein misfolding, aggrephagy, tumors, aggregates, proteostasis networks, chaperones

## Abstract

In normal physiological condition, the maintenance of cellular proteostasis is a prerequisite for cell growth, functioning, adapting to changing micro-environments, and responding to extracellular stress. Cellular proteostasis is maintained by specific proteostasis networks (PNs) to prevent protein misfolding, aggregating, and accumulating in subcellular compartments. Commonly, the PNs are composed of protein synthesis, molecular chaperones, endoplasmic reticulum (ER), unfolded protein response (UPR), stress response pathways (SRPs), secretions, ubiquitin proteasome system (UPS), and autophagy-lysosomal pathways (ALPs). Although great efforts have been made to explore the underlying detailed mechanisms of proteostasis, there are many questions remain to explore, especially in proteostasis regulated by the ALPs. Proteostasis out-off-balance is correlated with various human diseases such as diabetes, stroke, inflammation, hypertension, pulmonary fibrosis, and Alzheimer’s disease. Enhanced regulation of PNs is observed in tumors, thereby indicating that proteostasis may play a pivotal role in tumorigenesis and cancer development. Recently, inhibitors targeting the UPS have shown to be failed in solid tumor treatment. However, there is growing evidence showing that the ALPs play important roles in regulation of proteostasis alone or with a crosstalk with other PNs in tumors. In this review, we provide insights into the proteostatic process and how it is regulated by the ALPs, such as macroautophagy, aggrephagy, chaperone-mediated autophagy, microautophagy, as well as mitophagy during tumor development.

## 1. Introduction

Tumors are malignant diseases that are highly proliferative and unable to be controlled by the body. Over the past decades, many efforts have been made to understand the nature of tumors and to develop the strategies for cancer treatment. However, cancers still remain as a knotty problem threating human health. Before curing them, researchers must have a good knowledge of the molecular and cellular mechanisms underlying tumorigenesis and cancer development. During the last decades, researchers have identified several hallmarks of cancer, including sustaining proliferative signaling, escaping growth suppressors, counteracting cell death, enabling replicative immortality, inducing angiogenesis, promoting invasion and metastasis, acquiring genome instability, companying with inflammation, reprogramming of energy metabolism, evading immune destruction, and remodeling of the micro-environment [1]. Recently, reshaped proteostasis (protein homeostasis) has been shown to be a new hallmark in tumors, which would expand our knowledge about the nature of cancers [2].

Proteostasis is the maintenance of proteome homeostasis, thereby regulating protein translation, folding, trafficking, subcellular localization, and degradation [3]. The balance of proteostasis is important for normal cells to survive and function. Aberrant proteostasis can lead to loss-of-function diseases, such as cystic fibrosis and gain-of-toxic-function diseases, including neurodegenerative disorders like Alzheimer’s, Parkinson’s, and Huntington’s disease [4,5,6,7]. Recently, proteostasis also plays an important role in cancers [8]. Proteostasis in cancer is regulated by complicated proteostasis networks (PNs), including protein synthesis, molecular chaperones, endoplasmic reticulum (ER), unfolded protein response (UPR), stress response pathways (SRPs), ubiquitin proteasome system (UPS), autophagy-lysosomal pathways (ALPs), and secretions [9]. The UPS and molecular chaperones have been well elucidated to play a key role in the maintenance of proteostasis [10,11]. However, the detailed roles of the ALPs in the modulation of proteostasis in cancers have never been systematically summarized and fully elucidated.

In this review, we summarize the new concept of proteostasis in cancer, and its potential utilizations in cancer treatment. Besides, we also elucidate the ALPs and their regulatory manners in proteostasis. Our review aims to provide clues for researchers in this area to have a better understanding about the ALPs-regulated proteostasis and its connections between tumorigenesis and cancer development.

## 2. Proteostasis in Cancer

### 2.1. Proteostasis Needs to Be Balanced by Modulation of PNs

Under optimal non-physiological conditions, in which the protein concentration and temperature are low, a polypeptide, with specific amino acid sequence, can spontaneously form into a certain range of structures, which are relatively stable and dynamic functional. This state of a protein is commonly called the “native state” [12]. Besides which, stress-unfolded or *de novo*-synthesized polypeptides are difficult to refold because hydrophobic residues of the polypeptides are abnormally exposed to the aqueous phase, and for the intra-molecular stability, the unfolded hydrophobic residues may spontaneously form into improper beta sheets and incorrect inter-molecular ensembles, which are generally referred to as aggregates [13,14]. Furthermore, several misfolded polypeptides may synergistically have affinities with each other and result in aggregate-entrapped polypeptides, which may be cytotoxic and make the proteostasis imbalanced, thereby causing several disorders, such as neurodegenerative diseases and tumors [15].

While in normal cells, effective PNs composed of molecular chaperones, such as the USP and the ALPs, can eliminate proteotoxic aggregates. However, in the pathological state, the PNs are abnormal and lose or enhance the ability to eliminate these aggregates. So it’s important to modulate these regulators to maintain normal proteostasis.

### 2.2. Enhanced Regulation of PNs Is a New Hallmark of Cancer

There are many reasons why enhanced regulation of PNs is a new hallmark of cancer.

For instance, genome of cancer cell is instable and harbors numerous point mutations in DNA, some of which code mutated proteins that present significant folding challenges [16]. In addition, genomes of cancer cells also contains a lot of mutations in genes such as deletions, duplications, inversions, and translocations, and chromosomal mutations such as aneuploidy. Cells in over 90% of solid tumors harbor more than two copies of one or more chromosomes [17], which cause excessive protein synthesis and subsequent imbalanced proteostasis, also called proteotoxic crisis [18,19]. Therefore, cancer cells prefer producing misfolded proteins. Under this situation, cancer cells need some mechanisms that prevent such crises and alterations in regulation of proteostasis, thereby forming an abnormal proteome. For example, in melanoma cells, oncogene and aneuploidy drive dysregulations of proteostasis to impose a rewiring of proteostatic processes. Alterations of these proteostasis pathways act together with oncogenic pathways in melanoma cells to promote intrinsic adaptations for overcoming proteotoxic stress, reprogramming metabolic pathways, promoting metastasis, limiting response to therapy, and interacting with other cells in the micro-environment through release of regulatory factors or exosomes [20].

Secondly, cancers are ageing-related disorders. In the ageing cells, damaged and misfolded proteins are accumulated and promote proteostasis imbalance, thus impairing cellular function and tissue homeostasis [21]. In addition, in the PNs, there are also some SRPs, such as the nuclear factor erythroid 2-related factor 2 (Nrf2), which mobilizes genomic responses against oxidative or xenobiotic damage. Gradual accumulation of stressors during ageing promotes imbalanced PNs, which increase damaged and unstable proteomes and reduce DNA replication, fidelity, or repair, thereby promoting genomic instability and leading to tumorigenesis [22]. This is also the reason why ageing is related to neurodegenerative disorders.

In addition, cancers have different micro-environments, with fluctuations that may challenge the proteostasis. There are many initially stressful conditions that cancer cells face, such as hypoxia, acidosis, and low nutrient supply [8]. These stressful conditions make tumor cells break the balance of proteostasis to adapt to the new environment, thereby promoting the malignant hallmarks, such as invasiveness, cell metabolism reprogramming, avoidance of immune surveillance, resistance, and maintenance of stemness. Cells achieve proteostasis imbalance through a series of alterations of the PNs, which cause cancers to be PN-addicted. Enhanced proteostasis is also emerging as a new hallmark of cancers. Thus, targeting the abnormal PNs is likely to be a new strategy for cancer treatment.

## 3. Classical Proteostasis Networks in Cancer

**Figure 1:**
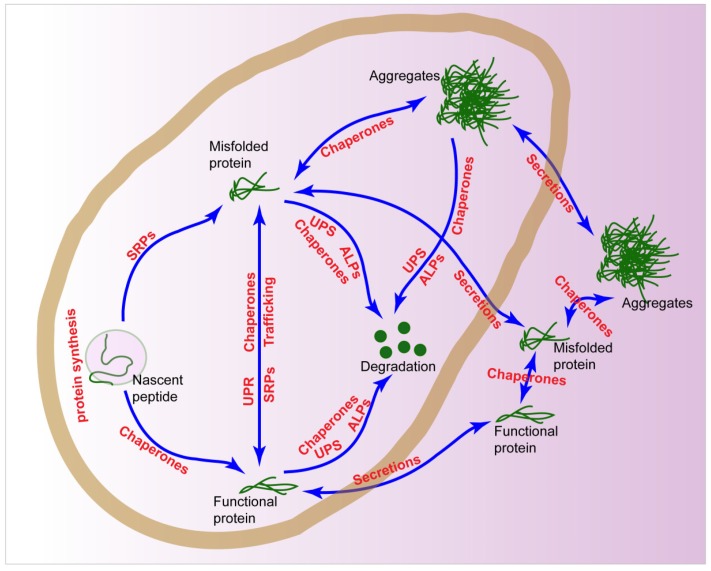
Proteostasis networks (PNs) in the cells. The proteostasis in a cell is regulated by protein synthesis, chaperones, unfolded protein responses (UPRs), stress response pathway (SRPs), ubiquitin proteasome system (UPS), autophagy-lysosomal pathways (ALPs), and secretions.

Proteostasis is commonly achieved through the coordinated action of PNs, including protein synthesis, trafficking modules, molecular chaperones, stress responses, degradations, and secretions (Figure 1). Actually, these PNs may cooperate with each other and form into intricate networks to regulate proteostasis in cancers.

### 3.1. Protein Synthesis

Protein synthesis *de novo* should match the need to produce new daughter cells and the rate of protein degradation [23]. Excessive synthesis is a cause for proteostasis imbalance [24], therefore the regulations of biosynthetic flux of proteins including amino acids availability, DNA transcription, and mRNA translation are important for proteostasis.

Usually, translation and ribosomes are the most important points of regulation for proteostasis. During translation, synthesis of a nascent protein is usually very slow and can even be stalled when it encounters a codon with low concentrations in cell, which is referred to a rare codon. This pause provides necessary time for an individual protein domain to properly fold before the production of the next domains, facilitating a multi-domain protein to be correctly folded [25]. Besides which, there is a narrow ribosome exit channel (width: 10 Å to 20 Å, length 80 Å) in the ribosome for the newly synthesized protein to form secondary and limited tertiary structures, such as alpha helix to exit into the cellular environment [26]. Meanwhile, the exit channel also inhibits premature folding by blocking large scale interactions within the protein.

It has been reported that ribosome reduction is an effective proteotoxic stress response [27]. In the normal cells, perturbations of the protein folding equilibrium induce an immediate translation reduction as an integral protein quality control that can be observed in the cytosol and in the organelles, such as the ER and mitochondria [24,28]. This machinery ensures a balanced proteostasis in the cells. In cancer biology, ribosomal biogenesis is an important process for cancer progression and is also an important target for tumor therapy [29]. Some regulators that regulate ribosomal biogenesis, such as fibroblast growth factor 13 (FGF13), miR-504, and p53, can support cancer cell survival by serving as an enabler for cancer cells to evade proteostasis stress triggered by oncogene activation [30].

### 3.2. Molecular Chaperones

Molecular chaperones are important regulators in the modulation of proteostasis. In protein synthesis and degradation, chaperones participate in two important processes, folding and unfolding, and assembly and disassembly [31]. Most of them are also known as heat shock proteins (HSPs) or stress proteins, and are a class of enzymes that have the ability to distinguish between unfolded, misfolded, and native protein conformers, then bind to exposed hydrophobic segments of substrate proteins to prevent the formation of stable, irreversible, non-functional, and amyloidogenic aggregates, thereby facilitating their appropriate folding [32]. They are ubiquitous, and are highly conserved from bacteria to eukaryotes [33]. One unfoldase chaperone can convert many misfolded or alternatively folded polypeptide substrates into transiently unfolded intermediates, which, once released, can spontaneously refold into low-affinity native products [15].

According to their molecular weights, they are divided into several groups [15,33], such as small HSPs (sHSPs), HSP40s/DnaJ, HSP60s/chaperonins/GroEL, HSP70s/HSP110s/DnaK, HSP90s/HtpG, HSP100s/ClpB, CCT (TRiC), and J-Protein families. The sHSPs function to prevent the aggregation of proteins and probable play a role in membrane homeostasis; the HSP40s function as HSP70 ATPase activators and perform intrinsic chaperone activity; the HSP60s participate in protein folding and aggregation, preventing bacterial and mitochondrial proteins; the HSP70s and HSP110s have organelle-specific variants and play important roles in folding of nascent protein, refolding of denatured proteins, and translocation across membranes; the HSP90s also have organelle-specific variants and contribute to protein maturation of steroid receptors, protein kinases, and other components of cellular signaling pathways; the HSP100s play essential roles in unfolding, solubilization of aggregates, and proteolysis; the CCTs (TRiC) contribute to folding of cytoskeleton components; the J-Proteins function as HSP70 and HSP110 targetases.

Pathological conditions in which chaperones become etiological or exhibit pathogenic factors are called chaperonopathies [34]. Chaperones are involved in several metabolic or molecular mechanisms of cancer cells and are implicated in the pathogenesis of various human cancers [35]. Recently, many chaperones, especially HSPs, have been demonstrated to be possible drug targets for cancer treatment, and this concept is referred to as chaperonotherapy [36,37,38]. For example, the effect of the Ras inhibitor, *S*-trans, trans-farnesylthiosalicylic acid (FTS, Salirasib^®^), is mediated by targeting Ras chaperones that serve as key coordinators for proper Ras folding and delivery. This drug has been successfully evaluated in clinical trials of cancer patients [39]. In addition, targeting HSP90 with 17-*N*-allylamino-17-demethoxygeldanamycin sensitizes glioblastoma to celastrol treatment [40].

However, since the protein target surfaces of the chaperones are relatively featureless, it’s very challenging to design or discover pharmacological chaperones for specific targets [41].

### 3.3. Trafficking Modules

Protein misfolding is a major reason for imbalanced proteostasis, and intracellular trafficking machinery also contributes to the clearance of misfolded proteins [42]. Therefore, the trafficking modules, including synaptic vesicle regulation, are important regulatory mechanisms in the modulation of proteostasis. Golgi apparatus (GA) play an important role in glycosylation, sulfation, and proteolysis of protein systems synthesized in the ER, as well as protein trafficking that modulates misfolding of protein aggregates. Fragmentation of the cisternae of GA is obviously observed in the Purkinje cells of the vermis and the cerebellar hemispheres of patients with Alzheimer’s disease [43].

The aberrant expression or activity of membrane receptors favors the malignancy of various cancer cell properties. There have been a lot of therapeutic drugs target the membrane receptors such as epidermal growth factor receptor (EGFR; Herceptin, Erbitux), tyrosine kinase (Iressa, Tarceva), CD20 (Rituxan, Arzerra), CD38 (Darzalex), and CD52 (Campath) [44]. Protein trafficking in cells plays a crucial role in the correct targeting of membrane receptors to the apical and basolateral surfaces, as well as to the adherent and tight junctions. Impaired availability or distribution of these receptors along the plasma membrane is not only associated with proteostasis and tumor progression, but also functions as emerging mechanism of resistance to targeted therapy in various cancers, such as colorectal cancer and lung cancer [45,46,47]. Particularly, protein trafficking displays a dual role in tumorigenesis and cancer development [48]. Firstly, defective delivery of proteins to the plasma membrane causes loss of cell polarity, which contributes to carcinogenesis in the early stage. Secondly, vesicle trafficking determines membrane dynamics that are crucial for the invasiveness of malignant cells. Therefore, a better understanding of the mechanisms of trafficking modules in cancers may provide clues for cancer treatment.

### 3.4. Unfolded Protein Response (UPR)

As a key site for lipid biosynthesis and folding of nascent transmembranes and secretory proteins, endoplasmic reticulum (ER) is the main dynamic organelle that participates into the regulation of proteostasis. Folding of proteins is maintained by careful homeostatic control of the environment within the ER lumen [49]. Under stress, the ER is unavailable for complete maturation of proteins, thereby breaking the balance of proteostasis to cause accumulated misfolded proteins, and triggering signaling sensors to activate the ER unfolded protein response (UPR) [50]. The UPR re-establishes proteostasis and protects cells from stress; however, under prolonged ER stress, the UPR can promote cell death [51].

The ER UPR is also termed the integrated stress response (ISR). In the ISR, four distinct serine and threonine protein kinases, including PERK, protein kinase R (PKR), general control nonderepressible 2 (GCN2), and heme-related eIF2α kinase (HRI), are involved and converge on the translation initiation factor eIF2α, resulting in phosphorylation at serine 51, which further induces a general inhibition of global protein synthesis, including protein chaperones and endoplasmic reticular structural proteins, and cell cycle arrest to alleviate ER stress [52]. Phosphorylation of eIF2α also promotes translation of activating transcription factor 4 (ATF4), glucose-related protein 78 (Grp78), and the expression of ATF4 target genes that ameliorate proteotoxic stress. However, it can also promote expression of another transcription factor, C/EBP-homologous protein (CHOP, also known as growth arrest and DNA damage-inducible protein 153, GADD153, or DNA damage inducible transcript 3, DDIT3) that induces apoptosis [53]. The different effects of UPR are dependent on the extent of the ER stress.

Recently, the UPR also emerge as a drug target for proteostasis-related disorders [54]. For example, aqueous extracts of *Paeonia suffruticosa* promotes reactive oxygen species (ROS), which induce ER stress, to impair mitochondrial proteostasis in pancreatic cancer cells [55].

### 3.5. Stress Responsive Pathways (SRPs)

A variety of cellular stresses, including oxidative stress [56], proteotoxic stress [57], ER stress [58], DNA damage stress [59], hypoxia stress [60], heavy metals and metalloids [61], and heat shock [62] can impair proteins and expose their hydrophobic domains which are buried within their interior in normal circumstances, thereby making them prone to aggregating, causing protein misfolding, aggregation, and proteotoxicity, thus breaking the balance of proteostasis. These processes crosstalk with the ER-UPR mentioned above, because ER is the main places where cells respond to these stresses. In addition, several other stress responsive pathways, including nuclear factor erythroid 2-related factor 2 (Nrf2), which mobilizes genomic responses against oxidative or xenobiotic damage, are also part of the PN [22].

Therefore, it is an important strategy to give stress to cancer cells, thereby inducing excessive proteotoxic stress that can kill cancer cells [63]. Radiotherapy and chemotherapy also give these stresses to cancer cells [64]. If the SRPs are blocked simultaneously, it will make the cancer cells more sensitive to these therapies [65].

### 3.6. Ubiquitin Proteasome System (UPS)

In eukaryotic cells, the UPS, the major mechanism by which proteins are degraded in the cytoplasm and nucleus, play key roles in the regulation of proteostasis [66]. In the UPS, proteins destined to be degraded are tagged by small protein ubiquitination in the lysine residues (usually K48U) with the help of E2 ubiquitin-conjugating enzyme and E3 ubiquitin ligase. Ubquitinated proteins bind to ubiquitin receptors and recruit the 26S proteasome, a multi-subunit protease, to link to and degrade them, recycling ubiquitin for future use.

In tumor cells, there is a perturbed proteome landscape, and the substrates of proteasome are usually misfolded or unassembled polypeptides, which make them rely more on the protein quality control (PQC) network than normal cells. The imbalanced PNs may sensitize cancer cells to drugs that target PQC regulators [67]. The proteasome inhibitors are promising anti-cancer drugs [68]. In multiple myeloma and mantle cell lymphoma, the proteasome inhibitors bortezomib and carfilzomib, which target PNs, have been shown to emerge as promising drugs to treat hematological malignancies [69,70,71,72]. However, in solid tumors, proteasome inhibitors have displayed little effect, even after many clinical trials, by virtue of more potent and specific proteasome inhibitors alone or in combination [73]. It has been shown that inhibition of proteasome induces a feedback regulation of its own expression and recovery of its activity, which make the UPS more plastic and causes the failure of proteasome inhibitors for cancer treatment [10].

In addition, some factors in the USP are aberrantly expressed in cancers and might be therapeutic targets for cancer treatment [74,75]. For example, COP9 signalosome subunit 6 (CSN6) is a regulator of the degradation of cancer-related proteins such as p53, c-myc, c-Jun, and EGFR through the UPS, and may be used as a potential therapeutic target in cancer [76]. The UPS also plays a central role in fine-tuning the functions of proangiogenic factors, such as vascular endothelial growth factor (VEGF), VEGF receptor 2 (VEGFR-2), and angiogenic signaling pathways, such as phospholipase C gamma 1 (PLCγ1) and phosphatidylinositol 3-kinase (PI3K)/AKT, and other non-VEGF angiogenic pathways [77]. However, much more work should be done to elucidate these factors in tumorigenesis and cancer development.

### 3.7. Secretions

In addition to above PNs, secretions also regulate the balance between intracellular and extracellular proteostasis. Under proteotoxic stress, the toxic aggregates can be secreted directly by exosomes or transporters to make sure of well-balanced intracellular proteostasis. When extracellular proteostasis is broken-down, some regulators can also be secreted outside of cells to interact with the aggregates and eliminate them to make sure of well-balanced extracellular proteostasis.

Commonly, excessive toxic proteins are secreted into extracellular spaces. For example, excess of tau can be secreted through membrane vesicles to avoid its toxicity [78]. In addition, exosomes also play important roles in controlling the decision between degradation and secretion, thus regulating the spread of neurotoxic protein aggregates and providing a mechanism for protein quality control [79].

Moreover, some regulators are also secreted outside the cells to modulate the extracellular proteostasis. For example, the UPR directly regulates extracellular proteostasis through upregulating and secreting the ER chaperone HSP40 ERdj3/DNAJB11 [80]. In the condition of ER stress, newly synthesized ERdj3 binds misfolded proteins on the ER and delivers them to binding-immunoglobulin protein (BiP), where they are chaperoned in the HSP70 cycle. However, when free BiP is lacking or becomes inefficient in eliminating the levels of misfolded proteins, the complex of ERdj3-misfolded proteins is co-secreted into the extracellular environment. In the extracellular space, ERdj3 binds the clients and substoichiometrically inhibits aggregates formation, thereby attenuating proteotoxicity of disease-associated toxic prion protein. Furthermore, ER stress-induced ERdj3 can also be secreted into the extracellular space to bind to misfolded proteins and toxic aggregates and attenuate them in the extracellular environment.

## 4. The Autophagy-Lysosomal Pathways in Proteostasis

In addition to those PNs mentioned above, recent studies also show that the ALPs may be a pivotal regulator for proteostasis [81]. The ALPs are major regulatory machineries in the degradation of long-lived proteins, deficits of which result in protein aggregation, the generation of toxic protein species, and accumulation of dysfunctional organelles, which are hallmarks of neurodegenerative diseases, systemic amyloidosis, prion disease, as well as some tumors [82].

**Figure 2:**
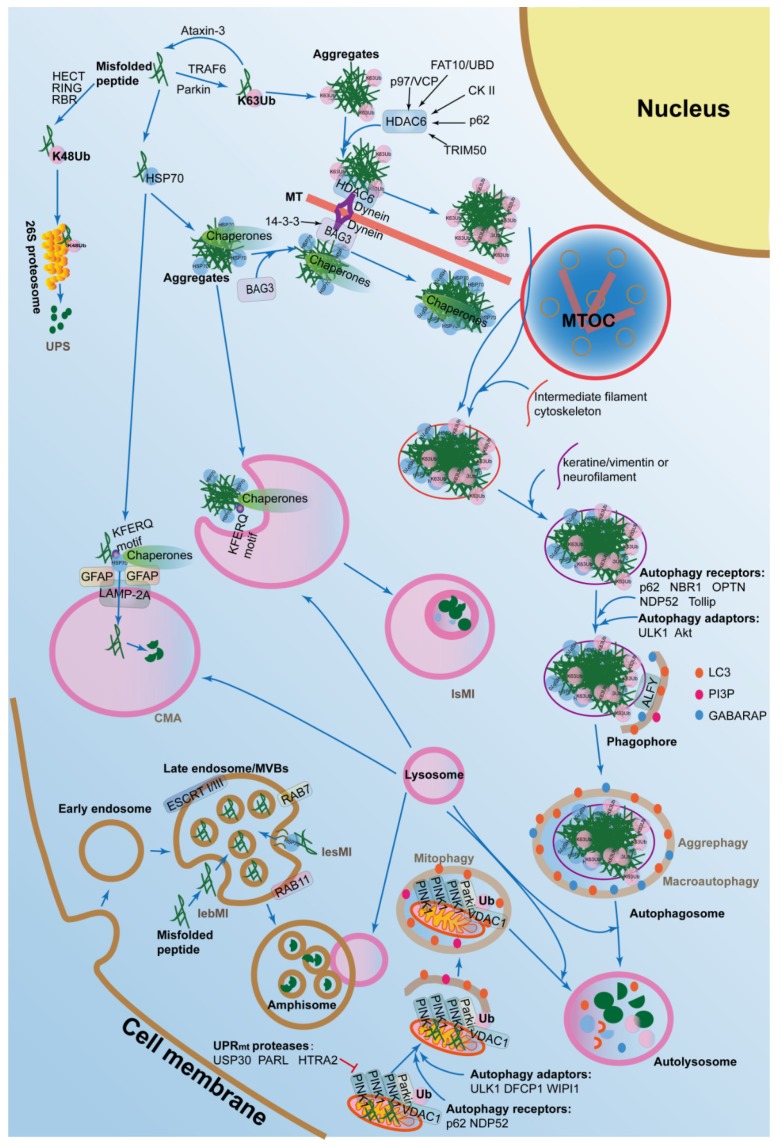
The autophagy-lysosomal pathways (ALPs) in the regulation of proteostasis. The ALPs are composed of macroautophagy, chaperone-mediated autophagy, aggrephagy, mitophagy, and microphagy. Abbrreviations: IsMI, lysosomal microautophagy; lesMI, late endosomal selective microautophagy; lebMI, late endosomal bulk microautophagy; CMA, chaperone-mediated autophagy; MT, microtubule; MTOC, microtubule organizing center; HECT, homologous to the E6-AP carboxyl terminus; RING, really interesting new gene; RBR, RING-betweenRING-RING; TRAF6, TNF receptor associated factor 6; p97/VCP, valosin containing protein; FAT10/UBD, ubiquitin D; CK II, casein kinase II; p62/SQSTM1, sequestosome 1; TRIM50, tripartite motif containing 50; HDAC6, histone deacetylase 6; BAG3, Bcl2-associated athanogene 3; GFAP, glial fibrillary acidic protein; LAMP-2A, lysosomal associated membrane protein 2; NBR1, neighbor of BRCA1 gene 1; OPTN, optineurin;NDP52, nuclear domain 10 protein 52; ULK1, Unc-51 like autophagy activating kinase 1; DFCP1, double FYVE-containing protein 1; WIPI1, WD repeat domain, phosphoinositide interacting 1; LC3, microtubule associated protein 1 light chain 3 alpha; PI3P, phosphatidylinositol 3-phosphate; GABARAP, gamma-aminobutyric acid receptor-associated protein; PINK1, PTEN induced putative kinase 1; Parkin, parkin RBR E3 ubiquitin protein ligase; MVBs, multivesicular bodies; ALFY, autophagy-linked FYVE-domain containing protein; VDAC1, voltage dependent anion channel 1; RAB7, Ras-related protein 7; RAB11, Ras-related protein 11; ESCRT I/III, endosomal sorting complex required for transport I/III; USP30, ubiquitin specific peptidase 30; PARL, presenilin associated rhomboid like; HTRA2, HtrA serine peptidase 2.

Autophagy, also called autophagocytosis, is a self-eating process that delivers cytoplasmic cargo to the lysosome. The ALPs mainly degrades protein aggregates that can be formed due to age-related, stochastic, non-enzymatic, post-translational modifications, as well as macromolecules, cytosolic portions, and entire organelles via lysosomes [83]. In mammalian cells, there are several forms of autophagy, including macroautophagy, microautophagy, mitophagy, chaperone-mediated autophagy (CMA), as well as aggrephagy (Figure 2), which are all induced by similar stimuli, such as extra environmental stress, nutrient starvation, oxidative stress, toxic stress, DNA damage, as well as infection, although mechanistic differences exist between the four groups.

### 4.1. Macroautophagy and Aggrephagy in Proteostasis

Macroautophagy is the main process of bulk protein degradation and is also the major core of the ALPs. There is a series of factors that participate in different stages of this biological process, including initiation, elongation, maturation, and delivery to the lysosome.

During the initiation stage, a crescent-shaped double-membrane structure called phagophore emerges. Unc-51 like autophagy activating kinase 1/2 (ULK1/2)-FAK family kinase-interacting protein of 200 kDa (FIP200)-autophagy related 13 (ATG13)-autophagy related 101 (ATG101) and PI3K-Akt-Vps34-Vps15-Beclin 1-Barker are two major complexes that are recruited to the phagophore assembly site (PAS). The former is inactivated by mechanistic target of rapamycin kinase (mTORC1) in normal situations, while activated by 5-AMP-activated protein kinase (AMPK) during starvation [84]. The latter is inhibited by Bcl-2, which forms a complex with Beclin 1 to antagonize the interaction of Beclin 1 with Vps34 [85,86]. PI3K also produce phosphatidylinositol 3-phosphate (PtdIns(3)P, PI3P) to concentrate at the surface of the phagophore and recruit other ATGs to the PAS to promote the formation of the autophagosome [87].

During the elongation stage, the phagophore elongates into phogosome by the aid of two ubiquitin-like conjugation systems, termed ATG4B-ATG3-ATG7-LC3 system and ATG5-ATG12-ATG16 system. The former cleaves microtubule associated protein 1 light chain 3 (LC3) into LC3-I by ATG4B and then cleaves LC3-I into LC3-II by ATG3 and ATG7 to conjunct to phosphatidylethanolamine (PE) [88]. The latter is crucial for the elongation of the pre-phogosomal structure and aid to the formation of LC-3II [89]. LC3-II finally localizes to the autophagosome and is a marker of autophagosome [90]. The resources of the autophagosome can be derived from multiple sub-cellular organelles with double-membrane structures, including cytoplasmic membrane, ER, Golgi apparatus, or mitochondrial membrane [91,92,93,94].

During the stages of maturation and lysosome fusion, the bubble-like autophagosome can move bidirectionally along microtubules via the aid of motor proteins, such as kinesin and dynein, and then form amphisome through fusing with endosome [95]. After this, amphisome fuses with lysosome to form autolysosome by the aid of several protein complexes, such as soluble NSF attachment protein receptors (SNAREs) [96]. Next, the autophagosome is digested by lysosomal enzymes.

Macroautophagy impairment in young cells induces the loss of proteostasis to promote cell senescence, with increased mitochondrial dysfunction and oxidative stress [97]. In the oxidative stress conditions, cellular proteomes are oxidated, damaged, or ubiquitinated, and then two cysteine residues, including C105 and C113 of sequestosome 1 (SQSTM1)/p62, are oxidized to promote its oligomerization and activate macroautophagy, thereby responding to oxidative stress to maintain proteostasis and increase cell survival [98]. In addition, in human fibroblasts, Ras-related protein RAB GTPase RAB18 enhances macroautophagy, depending on the expression of RAB3 GTPase activating protein catalytic subunit 1 (RAB3GAP1/2), which might act as RAB GDP-GTP exchange factors (GEFs) and stimulate the activity of the RAB GTPase. Therefore, RAB18 is relevant for the maintenance of proteostasis by eliminate the intracellular accumulation of ubiquitinated degradation-prone proteins [99]. These results indicate that macroautophagy plays an important role in the balance of proteostasis. Actually, removal of disease-linked aggresomes by using pharmacological upregulation of autophagy has been reported to slow down disease progression and to improve survival rates in many neurodegenerative animal models [100,101,102].

Aggrephagy refers to the biological process that degrades protein aggregates by macroautophagy in cells with imbalanced proteostasis. Protein aggregation is a continuous biological process in the cells. Some aggregates are required for normal functional processes in the cells, such as cellular defense, and they are modulated well. Importantly, it is reported that the presence of the smaller microaggregates dispersed throughout the cell are more toxic to the cell, compared to the large aggregates or inclusions. However, there are also some aggregates which are the result of protein misfolding in response to the intracellular or extracellular stresses. Aggregates are firstly ubiquitinated (K63U) by E3 ligase Parkin [103], and tumor necrosis factor receptor-associated factor 6 (TRAF6) [104] are specifically delivered to inclusion bodies by dynein-dependent retrograde transport on microtubules [105,106]. This process can be suppressed by Ataxin-3, an aggregation-associated deubiquitinating enzyme that cleaves away K63U in protein aggregates [107]. The microtubule-dependent inclusion bodies are called aggresomes. Then they recruit autophagic adaptors such as p62/SQSTM1, neighbor of BRCA1 gene 1 (NBR1), optineurin (OPTN), nuclear domain 10 protein 52 (NDP52), and Tollip, which directly interact with LC3 or the autophagosomal membrane to degrade the aggregates [106]. In summary, aggrephagy is dependent on the macroautophagic machinery.

Recently, histone deacetylase 6 (HDAC6) and Bcl-2-associated athanogene 3 (BAG3) have been shown to promote autophagosome formation in aggrephagy. Histone dacetylase HDAC6 can interact with the ubiquitinated protein aggregates and dynein of the dynein–dynactin motor complex, thereby promoting protein aggregates to load onto the microtubules for retrograde transport to the microtubule organizing center (MTOC) region [108]. However, protein aggregates with unanchored C-terminal ubiquitin chains from polyubiquitin linkages generated by ataxin-3 also can be recognized by HDAC6 [109]. Recently, many other regulators also modulate aggrephagy through interacting with HDAC6 at different levels. For example, casein kinase II (CKII) phosphorylates HDAC6 and increases its deacetylase activity [110]. SQSTM1/p62 also interacts with HDAC6 and increases its deacetylase activity [111]. Tripartite motif containing 50 (TRIM50), an E3 ubiquitin-ligase, can ensure the sequestration of the polyubiquitinated protein aggregates to the aggresome via associating with HDAC6, then TRIM50 also colocalizes and interacts with SQSTM1/p62 and increases its level [112]. The cytokine-inducible ubiquitin-like modifier FAT10 (also called ubiquitin D, UBD) interacts with HDAC6 and localizes to aggresomes, depending on an intact microtubule network under proteasome inhibition [113]. Molecular chaperone p97/valosin containing protein (VCP) also delivers ubiquitinated protein aggregates to HDAC6 under proteasomal stress. These evidences indicate that HDAC6-regulated aggrephagy is an alternative degradation pathway when the UPS is inhibited.

### 4.2. CMA and Aggrephagy in Proteostasis

In the CMA, substrate proteins contain a KFERO motif, which can be recognized by the chaperones, such as HSP70. Next, the HSPs-substrate complexes recruit and interact with lysosomal associated membrane protein 2 (LAMP-2A), the receptor on the lysosomal membrane, and induce LAMP-2A to assemble into multimers, which activate the transport function to deliver the cargo into lysosome after unfolding [114].

In fact, protein aggregates without ubiquitination can also be recognized and delivered to the MTOC region via the CMA. In the CMA, cytosolic chaperones recognize unfolding cytosolic proteins or aggregates selectively and deliver them to the lysosomal surface, where these proteins are internalized by virtue of a membrane translocation complex [115]. Therefore, the CMA-trafficked route provides an alternative mechanism for the sequesteration of the misfolded proteins or aggregates without ubiquitination into the autophagosome.

The major regulator, Bcl-2-associated athanogene 3 (BAG3), plays an independent regulatory model to promote autophagosome formation in the CMA-regulated proteostasis. Under proteotoxic stress, when proteasome is inhibited, BAG3 can be induced and can recognize HSP70 clients and interact with the dynein–dynactin motor complex by the aid of the 14-3-3 regulatory protein to deliver the HSP70 substrates along microtubules to the MTOC for sequestration into the aggresome [116,117].

### 4.3. Microautophagy in Proteostasis

Microautophagy is a process in which lysosome or late endosome directly engulfs cytoplasmic cargo, such as misfolded proteins. It is also triggered by starvation, nitrogen deprivation, and rapamycin treatment and proteostasis, just like those that drive macroautophagy. Microautophagy is divided into two types, which are termed lysosomal microautophagy (lsMI) and late endosomal microautophagy (leMI). The latter is also divided into two groups, including late endosomal selective microautophagy (lesMI) and late endosomal bulk microautophagy (lebMI) [118]. Protein cargo selection in the lesMI is mediated by the chaperone HSP70, whose cationic domain can electrostatically interact with negatively charged phosphatidylserine (PS) at the endosomal limiting membrane [119]. While in lebMI, the soluble cytosolic proteins are directly engulfed and delivered to the late endosomal vehicles, also called multivesicular bodies (MVBs). MVBs internalize the cytosolic cargos, which are subsequently digested by lysosome. The MVB formation relies on the endosomal sorting complexes required for transport (ESCRT) I and III systems [118] and RAB7, a small GTP-binding protein regulating the late endocytic pathway [120].

Recently, some aggregates in the cells were also reported to be regulated by microautophagy. For example, in the plants like *Arabidopsis thaliana*, the vacuolar membrane directly engulfs cytoplasmic flavonoid aggregates, such as anthocyanin aggregates by microautophagy. Next, a single membrane derived from the tonoplast surrounds the engulfed anthocyanin aggregates, which become free in the vacuolar lumen, just like an autophagic body. Eventually, the anthocyanin aggregates become densely packed, 3- to 10-μm diameter anthocyanin deposits, also called anthocyanin vacuolar inclusions (AVIs). However, there is neither endosomal/prevacuolar trafficking nor the autophagy ATG5 involving in this process, but it is promoted by the increase of cyanidin 3-O-glucoside derivative and the depletion of glutathione S-transferase, transparent testa 19 (TT19) [121]. Therefore, it may be a new mechanism for microautophagy in plant cells.

### 4.4. Mitophagy in Proteostasis

Mitophagy is a selective autophagic process that degrades damaged mitochondria via several major pathways, such as PTEN induced putative kinase 1 (PINK1)-Parkin pathway. In the initiation stage, the ubiquitin kinase PINK1 phosphorylates ubiquitin to activate the Parkin, an E3 ubiquitin ligase that builds ubiquitin chains on mitochondrial outer membrane proteins, such as voltage-dependent anion channel 1 (VDAC1). After ubiquitination, PINK1-Parkin recruit autophagy receptors, such as NDP52 and optineurin [122] or p62/SQSTM1 [123] to the damaged mitochondria, where they recruit the autophagic adaptor ULK1, double FYVE-containing protein 1 (DFCP1), and WD repeat domain, phosphoinositide interacting 1 (WIPI1), to focal spots proximal to mitochondria and cleave LC3 to trigger mitophagy. Mitophagy is also regulated by some regulations, such as 5ʹAMP-dependent kinase (AMPK)-dependent phosphorylation of ULK1 at Ser555 [124].

There are at least 1158 mitochondrial proteins in humans, and 99% of them are encoded by the nuclear genome [125]. Most of the proteins are imported into the mitochondria in an unfolded state, and are properly folded by various chaperones within the mitochondrial compartments to exert their function. To maintain proteostasis in the mitochondria, there are usually various proteases and chaperones in mitochondria [126]. Recently, it was reported that mitophagy is essential for the balance of proteostasis to maintain health and ageing [127]. Mitophagy combined with mitochondrial unfolded protein response (UPR^mt^) is also important for maintenance of mitochondrial proteostasis to reduce amyloid-β proteotoxicity, the main cause of the Alzheimer’s disease [128]. Besides which, impairment of mitophagy in skeletal muscle results in accumulation of damaged or dysfunctional mitochondria, thereby inducing the loss of mitochondrial proteostasis and ageing [129]. Exercise exerts many systemic health benefits against ageing through mitophagy to improve mitochondrial proteostasis in skeletal muscle [130].

## 5. Proteostasis Regulated by the ALPs Are Important for Tumor Malignancy

It is well known that autophagy play essential roles in the modulation of pathological development of various tumors [131,132], including lymphoma [133], glioblastoma [134], neuroblastoma [132], melanoma [86], gastric cancer [135], colorectal cancer [136], and breast cancer [137]. However, little is known about proteostasis regulated by autophagy in tumors. During the last decade, growing evidence shows that proteostasis in cancer cells is regulated by autophagy.

Interestingly, some essential components of the autophagy directly interact with proteins determined to be degraded, thus regulating proteostasis in tumors. Mevalonate pathway, a metabolic process that has potential implications for cancer, regulates basal autophagic flux through geranylgeranylating the small GTPase RAB11, thereby influencing proteostasis to control cell size and cell growth [138].

There are some evidences showing that proteostasis regulated by autophagy plays essential roles in multiple myeloma [139,140]. For example, peIF4E silence or inhibition attenuates its targets, such as c-Myc, cyclin D1, and breaches proteostasis via inhibiting Akt, which is a major regulator in autophagy pathways [139]. Besides which, autophagic cargo receptor and adapter protein, SQSTM1/p62, is shown not only to synergize with the proteasome to maintain proteostasis, but also mediates a plastic adaptive response to proteasome inhibitors in multiple myeloma [140].

In human pancreatic cancer (PC) cells, simultaneous inhibition of the ubiquitin-proteasome system and autophagy by withaferin-A (WA), the biologically active withanolide extracted from *Withania somnifera*, enhances apoptosis induced by ER stress aggravators both *in vitro* and *in vivo* [141]. These findings indicate that suppression of 2 PNs renders PC cells vulnerable to ER stress, which may provide clues for new therapeutic combinations for PC.

In addition to macroautophagy, mitophagy and mitochondrial proteostasis also participate into tumorigenesis or cancer progression. Proteases such as ubiquitin specific peptidase 30 (USP30), presenilin associated rhomboid like (PARL), and HtrA serine peptidase 2 (HTRA2) in the UPR^mt^ can interact with mitophagic regulator PINK1-Parkin, and then inhibit mitophagy and maintain mitochondrial proteostasis in cancers [142]. In addition, accumulation of misfolded proteins in the mitochondria induces proteotoxic stress, which activates mitophagy and SIRT3 to promote the UPR^mt^ for cancer cells to adapt to proteotoxic and mitochondrial stress [143].

## 6. The ALPs Have Crosstalk with Other PNs in the Regulation of Proteostasis in Cancers

The maintenance of proteostasis needs all the PNs to cooperate. Therefore, the ALPs-regulated proteostasis usually crosstalk with the other PNs in cancer cells.

Firstly, it is well known that the CMA is dependent on the chaperones. So the ALPs are highly interwined with chaperones in the regulation of proteostasis in tumors.

Secondly, the ALPs crosstalk in protein synthesis. For example, mTORC1 can activate NRF1 to enhance protein synthesis and inhibit autophagy, thereby promoting the production of more proteasomes. Increase in proteasome levels facilitates both the maintenance of proteostasis and the recovery of amino acids [144].

Thirdly, the ALPs crosstalk with the SRP. For example, mTORC1, a major regulator in autophagy, is also shown to regulate protein folding and proteasomal degradation as well, thus playing a prominent role in proteostasis. Mechanically, mTORC1 reacts to diverse stresses, including energetic or metabolic stress, genotoxic stress, oxidative stress, osmotic stress, ER stress, proteotoxic stress, and psychological stress, thus playing a regulatory role in cellular proteostasis [145].

Fourthly, the ALPs crosstalk with the UPR. For example, ER stressors can trigger the UPR and increase a number of the UPR effector mechanisms, including autophagy [49], which subsequently induces cell survival or death [51]. As a non-canonical cargo receptor, cell-cycle progression gene 1 (CCPG1), inducible by the unfolded protein response, can directly bind to core autophagy proteins via an LIR motif to ATG8, and independently via a discrete motif, to FIP200, thereby facilitating ER-phagy (autophagy of the ER) and keep balance of ER proteostasis to protect against tissue injury of the exocrine pancreas [146].

Finally, the ALPs crosstalk with the UPS. For example, SQSTM1/p62, an essential component of the autophagic reserve, synergizes with the proteasome to maintain proteostasis and determines proteasome inhibitor (PI) susceptibility in multiple myeloma cells [140]. Mechanically, under basal conditions, SQSTM1/p62-dependent autophagy constitutively disposes of substantial amounts of ubiquitinated proteins, thus alleviating the degradative burden on the proteasome. Inhibition of SQSTM1/p62 significantly sensitizes PI-induced protein aggregation and cell death. While under proteasome stress, SQSTM1/p62 *de novo* expression is selectively enhanced and its vast endogenous interactome are also reset in myeloma cells, thus promoting SQSTM1/p62 to divert from signaling partners to associate with ubiquitinated proteins [140].

In fact, the ALPs crosstalk with other PNs in a complex manner. More and more evidences have shown that the PNs in cancer cells do not work alone. Actually, there are complex PNs that regulate the proteostasis in cancers. They often crosstalk with each other in a complementary manner. For example, Withaferin A inhibits the UPS and the ALPs, thus causing accumulation of ubiquitinated proteins, which in turn led to ER UPS-mediated proteotoxicity in human breast cancer cells [147]. Similarly, simultaneous inhibition of the UPS and the ALPs by genetic or therapeutic inhibition enhances ER stress aggravator-mediated cell apoptosis in human pancreatic cancer cells [141]. Loss of HSP83, the *Drosophila* ortholog of human chaperone HSP90 (heat shock protein 90), suppresses proteasomal activity and upregulates caspase-dependent compensatory autophagy [148]. In addition, some regulators also regulate proteostasis via a complex PNs. For example, BAG3 can either promote the activity of molecular chaperones, sequester and concentrate misfolded proteins, initiate autophagic disposal, or balance transcription, translation, and degradation [149].

## 7. Concluding Remarks

The balance of proteostasis is very important for normal cells to survive and enhanced regulation of PNs is a novel hallmark of cancer cells. Abnormal proteostasis may be the cause of many diseases, including neurodegenerative diseases, systemic amyloidosis, prion disease, cystic fibrosis, lysosomal storage disorders, and tumors [150]. Furthermore, imbalanced proteostasis also has a connection with obesity and ageing, which are tightly correlated with the initiation and progression of tumors and neurodegenerative disorders. Current studies have revealed the connections between imbalanced proteostasis with neurodegenerative diseases. However, the relationship between abnormal proteostasis with tumors still remains to be summarized.

As mentioned above, cancer cells commonly have enhanced PNs, which help them to eliminate excessive aggregates and misfolded proteins to avoid proteotoxicity. Thus, blocking of PNs is a promising strategy for cancer treatment. Previous reports have showed that some cancer cells are susceptible to chaperone inhibitors and proteasome inhibitors that disrupt proteostasis. Importantly, proteasome inhibitor bortezomib (PS-341), a dipeptide boronic acid, is the first clinical drug used for treatment of multiple myeloma [151]. Actually, there is also an alternative pathway for aggregate degradation in tumor cells, which is termed the ALPs. Besides which, autophagy is also a target for promising treatment of various tumors [152]. Maybe there are some clues for treating cancer in the understanding of the mechanism of the ALPs-regulated proteostasis. Therefore, in this article, we summarize the mechanisms of different kinds of ALPs, including macroautophagy, aggrephagy, microautophagy, CMA, and mitophagy in the regulation of proteostasis and their effects in cancers. Although the ALPs are also important for cancer cells to maintain proteostasis, they can never be inhibited alone for cancer treatment, because the UPS will be an alternative way for aggregates to degrade [153]. Therefore, the best way is to impair both routes that eliminate the aggregates in cancer cells. Just like radiotherapy and chemotherapy induces DNA damage in cancer cells with many DNA mutations, proteostasis-related therapy also induces more proteotoxic aggregates and misfolded proteins in cancer cells with many misfolded proteins and aggregates. In conclusion, it is a promising strategy to block both the UPS and the ALPs so as to induce proteotoxic aggregates and lead to cell death in cancers.
